# Safety and short-term outcomes of gastrectomy after preoperative chemotherapy plus immunotherapy versus preoperative chemotherapy: a retrospective cohort study

**DOI:** 10.1186/s12885-022-10272-5

**Published:** 2022-12-13

**Authors:** Yinkui Wang, Xiaokang Lei, Fei Shan, Shuangxi Li, Yongning Jia, Rulin Miao, Kan Xue, Zhemin Li, Jiafu Ji, Ziyu Li

**Affiliations:** 1 Gastrointestinal Cancer Center, Peking University Cancer Hospital and Institute, 52 Fucheng Road, Haidian District, 100142 Beijing, China; 2 Key Laboratory of Carcinogenesis and Translational Research (Ministry of Education/Beijing), Peking University Cancer Hospital and Institute, Beijing, China

**Keywords:** Safety, Gastrectomy, Preoperative, Chemotherapy plus immunotherapy, Chemotherapy

## Abstract

**Background:**

The safety and short-term outcomes of gastrectomy after preoperative chemotherapy plus immunotherapy (PCIT) versus preoperative chemotherapy (PCT) in patients with advanced gastric cancer (AGC) remain unclear. This study was conducted to compare the safety and short-term efficacy of PCIT with those of PCT in patients with AGC.

**Methods:**

We retrospectively reviewed the data of patients with AGC who received PCIT or PCT at Peking University Cancer Hospital and Institute Gastrointestinal Cancer Center I between January 2019 and June 2021. The clinical characteristics were recorded, and short-term oncological outcomes were compared. Independent t tests, Mann‒Whitney U tests, chi-square tests, and Fisher’s exact tests were used to calculate differences. The correlation analyses were performed using Pearson correlation. All *p* values were two-sided, and a *p* value < 0.05 was considered statistically significant. All the above statistical analyses were conducted by the SPSS version 24.0 software package (IBM Corp., Armonk, NY, USA).

**Results:**

A total of 162 AGC patients were included in this study, including 25 patients who received PCIT and 137 patients who received PCT. There were no significant differences in preoperative treatment-related adverse events (TRAEs) between the PCIT group and the PCT group (*p* = 0.088). Compared with the PCT group, the PCIT group had comparable postoperative functional recovery, with no significant differences in terms of time to first aerofluxus (*p* = 0.349), time to first defecation (*p* = 0.800), time to liquid diet (*p* = 0.233), or length of stay (*p* = 0.278). No significant differences were observed in terms of postoperative complications (*p* = 0.952), postoperative pain intensity at 24, 48, or 72 h (*p* = 0.375, *p* = 0.601, and *p* = 0.821, respectively), or postoperative supplementary analgesic use between the two groups (*p* = 0.881). In addition, the postoperative complication rate was 33.3% following laparoscopic approaches and 31.2% following open approaches in the PCIT group, with no significant difference (*p* = 1.000).

**Conclusion:**

In patients with AGC, gastrectomy with D2 or D2 + lymphadenectomy after PCIT had comparable short-term oncological outcomes to PCT.

**Supplementary Information:**

The online version contains supplementary material available at 10.1186/s12885-022-10272-5.

## Introduction

Gastric cancer (GC) is one of the most common malignant tumors of the digestive system. According to the latest statistical data of GLOBOCAN, there were more than one million new cases of GC worldwide in 2020, resulting in an estimated 769,000 deaths; these data imply that one in every 13 deaths globally is due to GC, with CG ranking fifth for incidence and fourth for mortality globally [1]. A high incidence of GC is observed in China, with 403,000 new cases and 291,000 deaths annually. Compared with other East Asian countries with a high incidence of GC, most of the patients in China are diagnosed with AGC [2]. At present, the treatment for AGC is still a major challenge due to its high rates of recurrence and metastasis [3]. PCT followed by gastrectomy has been proven to be a safe and effective method [4–10] and is widely used for the treatment of AGC [11–14], with better rates of micrometastasis eradication and R0 resection.

As a new promising approach to cancer therapy, immunotherapy has also gradually been used for the treatment of GC. According to the results of several previous studies [15–17], the U.S. Food and Drug Administration (FDA) and China National Medical Products Administration (NMPA) have approved immunotherapy with or without chemotherapy for the treatment of patients with metastatic or unresectable GC. Based on the great success of immunotherapy for metastatic or unresectable GC patients, some experts have explored the application of preoperative immunotherapy in resectable or potentially resectable AGC. Some case reports have suggested that preoperative immunotherapy followed by gastrectomy for the treatment of AGC patients is effective and safe [18–20]. Previous studies have shown that preoperative immunotherapy might be associated with increased wound complications in head and neck cancer [21, 22]. However, one study indicated that esophagectomy was not associated with an increased risk of perioperative morbidity or mortality in patients with locally advanced esophageal cancer treated with neoadjuvant chemoradiation plus immunotherapy [23]. How PCIT followed by gastrectomy compares with PCT for the treatment of AGC in terms of safety and efficacy remains unknown. Thus, we conducted this study to compare the safety and short-term efficacy of gastrectomy after PCIT with those of PCT in patients with AGC.

## Patients and methods

### Inclusion and exclusion criteria

Patients with resectable or potentially resectable AGC who received chemotherapy plus immunotherapy or chemotherapy before surgical treatment were included in this study. All included patients were treated at Peking University Cancer Hospital and Institute Gastrointestinal Cancer Center I between January 2019 and June 2021. Patients aged between 18 and 80 years old with a Karnofsky performance scale score higher than 70 were eligible. In addition, patients were excluded if they had (1) other malignancies, (2) a history of upper abdomen operation other than laparoscopic cholecystectomy, (3) medical conditions requiring emergency surgeries, (4) a history of receiving any radiotherapy or antiangiogenesis treatment before surgery, or (5) a history of thoracotomy. The study was approved by the Ethics Committee of Beijing Cancer Hospital (No. 2017XJS07), and all eligible participants provided written informed consent.

### Preoperative treatment strategies

The determination of treatment routines was managed by the multidisciplinary team (MDT). The choice of treatment strategy, including PCIT or PCT, was decided through shared decision-making between patients and doctors, with consideration of gastroscopic biopsy pathological immunohistochemical results, such as microsatellite status, tumor mutational burden (TMB), EBER status, programmed death-ligand 1 combined positive score (PD-L1 CPS) and other results. The doctors informed patients that PCT was the standard treatment. If the patients had one or more of the following indicators, such as microsatellite instability-high (MSI-H), TMB-high, EBER-positive, or PD-L1 CPS ≥ 5, PCIT was strongly recommended. If the patients and their relatives agreed, PCIT was employed; otherwise, PCT was employed. The surgical approach, either open gastrectomy or laparoscopic gastrectomy, was chosen according to the preferences of each patient after sufficient explanation of the advantages and risks of both approaches. If a patient could not decide on the surgical approach, the chief surgeon decided based on actual operative circumstances. In each group, the range of gastrectomy and the extent of lymphadenectomy were both chosen based on the Japanese Gastric Cancer Treatment Guidelines 2018 (5th edition) [24] and the Japanese Classification of Gastric Carcinoma: 3rd English Edition guidelines [25]. Laparoscopic exploration was performed to determine whether there was adjacent organ invasion and peritoneal dissemination prior to the operation.

### Surgical techniques

An incision of approximately 20–25 cm in length was made from the falciform process to the periumbilical area in patients who chose open approaches. Laparoscopic surgery was performed using five trocars, including one 12-mm trocar below the umbilicus for the camera, two five-mm trocars that were placed in the right anterior axillary below the costal margin level and the left clavicular midline at the umbilical level, and two additional 12-mm trocars that were placed in the right clavicular midline at the umbilical level and the left anterior axillary below the costal margin. The main reconstruction methods included esophagogastrostomy, double-tract reconstruction, Roux-en-Y, uncut Roux-en-Y, and standard Billroth I or Billroth II gastrojejunal anastomosis with Braun’s anastomosis. The chief surgeon chose a suitable reconstruction method depending on the actual operative conditions. These procedures were performed by a single surgical team with extensive experience in both open and laparoscopic procedures to treat GC. Conversion to open surgery was performed when the surgeon considered an open approach to be necessary for a particular surgical situation, such as in the case of dense peritoneal adhesions, pneumoperitoneum intolerance, severe hemorrhaging during operation, and extensive tumor invasion. During the perioperative periods, all the patients were managed by a standard clinical scheme. Discharge was recommended when the patient tolerated more than two days of a soft diet without abdominal pain or fever.

### Data collection and outcome measurements

Demographic characteristics, including age, sex, weight, height, comorbidities, American Society of Anesthesiology (ASA) scores, and pathological characteristics, were collected from all included patients. Body mass index (BMI) was calculated from weight and height for each patient. Enhanced abdominal computed tomography (CT) was conducted before and after preoperative treatment in each patient to evaluate the preoperative treatment response according to Response Evaluation Criteria in Solid Tumors (RECIST) version 1.1 [26]. The overall response rate (ORR) was defined as the percentage of patients who achieved complete response (CR) or partial response (PR). The disease control rate (DCR) was defined as the ORR plus the rate of stable disease (SD). Pathological and clinical stages were determined according to the 8th edition of the International Union Against Cancer (UICC) TNM classification [27]. In addition, tumor regression grade (TRG) was also used to evaluate the response to preoperative therapy based on the NCCN Clinical Practice Guidelines in Oncology for Gastric Cancer (version v3.2015) [28].

The length of incision, operation time, and estimated blood loss were collected. Postoperative recovery-relevant indices, including time to first aerofluxus, time to first defecation time, time to liquid diet, time to drainage removal, and length of hospital stay after surgery, were also recorded. Furthermore, the number of retrieved and metastatic lymph nodes, long and short tumor diameters, proximal margin length of the tumor, and distal margin length of the tumor, which were measured on fresh surgical tissue specimens, were recorded. The visual analog scale (VAS) [29, 30] was used to assess postoperative pain intensity at 24, 48, and 72 h after surgery. The duration of intravenous patient-controlled analgesia (IV-PCA) use and the supplementary analgesic dose were recorded in each patient. The supplemental analgesic dose after surgery was converted to oral morphine equivalents (OMEs) [31]. Postoperative complications were defined as conditions that occurred during the hospital stay following surgery and were graded using the Clavien‒Dindo classification system [32, 33]. Severe complications were defined as Clavien‒Dindo grade III or greater. Postoperative mortality was defined as death that occurred within 30 days after initial surgery, regardless of cause.

### Statistical analysis

In the case of continuous variables, the data are displayed as the means and standard deviations, and in the case of categorical variables, the data are displayed as proportions. For high-skew data, the results are presented as the medians (interquartile ranges, IQRs). Independent t tests, Mann‒Whitney U tests, chi-square tests, and Fisher’s exact tests were used to determine differences between the two groups’ baseline data and outcomes. The correlation analyses were performed using Pearson correlation. All *p* values were two-sided, and a *p* value < 0.05 was considered statistically significant. All the above statistical analyses were conducted by the SPSS version 24.0 software package (IBM Corp., Armonk, NY, USA).

## Results

### Baseline demographic and clinicopathologic characteristics

A total of 162 patients were included in the study, including 25 patients in the PCIT group and 137 patients in the PCT group. A significant difference was observed in age between the two groups [median (IQR) age of 58 (37.5, 67) years in the PCIT group vs. 64 (54, 69) years in the PCT group, *p* = 0.035]. The other demographic and clinicopathologic characteristics, including sex, BMI, ASA score, comorbidities, abdominal surgery, tumor long and short diameters, clinical stage, RECIST criteria, preoperative regimen, preoperative cycle, Borrmann type, adverse events, surgery approach and extent of gastrectomy, were comparable between the two groups. The detailed demographic and clinicopathologic characteristics of the two groups are provided in Table 1.

**Table 1:** Demographic and clinicopathologic characteristics in the PCIT group and PCT group

Characteristics	PCIT group (*n* = 25)	PCT group (*n* = 137)	*p*-value
Age	58(37.5,67)	64(54,69)	0.035
BMI	22.82 ± 0.63	23.49 ± 0.30	0.375
Gender			0.992
Male	19(76.0)	104(75.9)	
Female	6(24.0)	33(24.1)	
ASA score			0.232
1	2(8.0)	4(2.9)	
2	23(92.0)	133(97.1)	
Comorbidity			0.429
No	9(36.0)	61(44.5)	
Yes	16(64.0)	76(55.5)	
Abdominal surgery			0.539
No	20(80.0)	118(86.1)	
Yes	5(20.0)	19(13.9)	
Diameter in long axis(cm)^a^	4(3,6)	4.75(3,6)	0.625
Diameter in short axis(cm)^b^	2.5(2.0,4.0)	3.0(2.0,5.0)	0.164
cTNM stage			0.570
II	0(0)	6(4.4)	
III	17(68.0)	81(59.1)	
IV	8(32.0)	50(36.5)	
ycTNM stage			0.461
I	0(0.0)	3(2.2)	
II	1(4.0)	14(10.2)	
III	19(76.0)	85(62.0)	
IV	5(20.0)	35(25.5)	
ypTNM stage			0.011
0	5(20.0)	10(7.3)	
I	6(24.0)	14(10.2)	
II	8(32.0)	45(32.8)	
III	3(12.0)	59(43.1)	
IV	3(12.0)	9(6.6)	
Borrmann type			0.188
I	0(0)	6(4.4)	
II	7(28.0)	18(13.1)	
III	17(68.0)	103(75.2)	
IV	1(4.0)	10(7.3)	
The degree of malnutrition			0.027
Normal	22 (88.0)	135 (98.5)	
Mild malnutrition	2 (8.0)	1 (0.7)	
Moderate malnutrition	1 (4.0)	0 (0.0)	
Severe malnutrition	0 (0.0)	1 (0.7)	

^a^Diameter in long axis (cm): one case data in the chemotherapy group was missing

^b^Diameter in short axis (cm): one case data in the chemotherapy group was missing

### Preoperative therapy regimen, response rate, and related adverse effects

The routine regimens used for PCT were one-, two- or three-drug regimens based on fluorouracil. The treatment regimens used for PCIT included the use of anti-PD-1 antibodies or anti-CTLA-4 antibodies in conjunction with the above chemotherapy regimens.

The ORR in the PCIT group was 36.0%, while in the PCT group, it was 18.2%. The DCR was 92% in the PCIT group and 93.4% in the PCT group. There were 16 (64.0%) patients in the PCIT group and 95 (69.3%) patients in the PCT group who received four or fewer cycles of preoperative treatment. Data were missing for 6 patients, and TRAEs occurred in 71.6% of all included patients (116/162). Severe adverse events (SAEs), grade 3 or 4 based on the National Cancer Institute Common Terminology Criteria for Adverse Events (CTCAE) version 5.0 guidelines, occurred in 12.96% of all patients (21/162). The preoperative treatment regimens, TRAEs, response rates, and treatment cycles for the two groups are shown in Table 2.

**Table 2:** Preoperative treatment regimens, response rates, and treatment related adverse effects in the PCIT group and PCT group

Preoperative treatment characteristics	PCIT group (*n* = 25)	PCT group (*n* = 137)	*p*-value
RECIST Criteria (version 1.1)^a^			0.151
Partial response	9(36.0)	25(18.2)	
Stable disease	14(56.0)	103(75.2)	
Progressive disease	1(4.0)	2(1.5)	
Tumor regression grade (TRG)			0.030
0	5 (21.7)	10 (7.6)	
1	3 (13.0)	15 (11.5)	
2	12 (52.2)	52 (39.7)	
3	3 (13.0)	54 (41.2)	
Preoperative treatment regimen			0.074
One-drug regimen	1(4.0)	0(0)	
Two-drug regimen	21(84.0)	109(79.6)	
Three-drug regimen	3(12.0)	28(20.4)	
Preoperative treatment cycle			0.597
≤4	16(64.0)	95(69.3)	
>4	9(36.0)	42(30.7)	
Adverse event(AE)^b^			0.088
Grade 0	11(45.8)	29(22.0)	
Grade 1	5(20.8)	48(36.4)	
Grade 2	7(29.2)	35(26.5)	
Grade 3	1(4.2)	16(12.1)	
Grade 4	0(0)	4(3.0)	

^a^RECIST Criteria (version 1.1): There was one case missing data in the chemotherapy plus immunotherapy group and seven cases missing data in the chemotherapy group

^b^Adverse event (AE): There was one case missing data in the chemotherapy plus immunotherapy group and five cases missing data in the chemotherapy group

### Surgical and pathological characteristics

One patient (4.0%) in the PCIT group underwent proximal gastrectomy (PG), and nine patients (6.6%) in the PCT group underwent PG. Twelve (48%) patients in the PCIT group and 78 (56.9%) patients in the PCT group underwent total gastrectomy (TG). Twelve (48%) patients in the PCIT group and 50 (36.5%) patients in the PCT group received distal gastrectomy (DG). There was no significant difference in the range of gastrectomy between the two groups (*p* = 0.595). All patients received D2 or D2 + lymphadenectomy with R0 resection. Combined resections were performed in 23 of all patients [1 (4.0%) patient vs. 22 (16.1%) patients; PCIT vs. PCT group; *p* = 0.132)] because of infiltration of adjacent organs or gallbladder issues, such as gallbladder stones or history of cholecystitis. Resected organs included gallbladder, diaphragm, partial colon or mesentery, partial liver, spleen, partial pancreas, and ovary. Nine patients (36%) in the PCIT group received laparoscopic approaches (*p* = 0.647), while in the PCT group, 56 patients (40.9%) received laparoscopic approaches. The approach for one patient (4%) in the PCIT group was converted to an open approach due to bleeding. The approaches for five patients (3.6%) in the PCT group were converted to open approaches due to bleeding (two patients), tumor adhesion (two patients), and obesity (one patient). No statistically significant difference was found between the two groups in the rate of conversion to an open approach (*p* = 1.000). The proximal margin length and distal margin length of tumors were not significantly different between the two groups [median (IQR) proximal tumor margin length 4 (2, 6.88) cm in the PCIT group vs. 3 (1.5, 6.0) cm in the PCT group, *p* = 0.287; median (IQR) distal tumor margin distance 4.5 (2, 9.63) cm vs. 7 (3.30, 11.50) cm, *p* = 0.096]. Comparable numbers of lymph nodes (LNs) were retrieved in both groups, with the median (IQR) number of LNs per patient being 37 (37, 46.5) in the PCIT group and 33.0 (27, 44.5) in the PCT group. However, there were significantly more metastatic LNs in the PCT group than in the PCIT group [median (IQR) 2 (0, 8) vs. 2 (0, 3.5); *p* = 0.014]. Neither the number of retrieved LNs nor the number of metastatic LNs at each station was significantly different between the two groups (*p* = 0.287 and *p* = 0.096, respectively). The TRG data were as follows: PCIT group: TRG0, five patients (21.7%); TRG1, three patients (13.0%); TRG2, 12 patients (52.2%); and TRG3, three patients (13.0%); PCT group: TRG0, ten patients (7.6%); TRG1, 15 patients (11.5%); TRG2, 52 patients (39.7%); and TRG3, 54 patients (41.2%). The details are presented in Table 3

**Table 3:** Comparison of surgical characteristics between the PCIT group and PCT group

Tumor parameters	PCIT group (*n*=25)	PCT group (*n*=137)	*p*-value
Surgical approaches			0.647
Open approach	16(64.0)	81(59.1)	
Laparoscopic approach	9(36.0)	56(40.9)	
Conversion to open from laparoscopic	1(4.0)	5(3.6)	1.000
Combined organ resection			0.132
No	24(96.0)	115(83.9)	
Yes	1(4.0)	22(16.1)	
Gastrectomy range			0.595
Proximal gastrectomy	1(4.0)	9(6.6)	
Distal gastrectomy	12(48.0)	50(36.5)	
Total gastrectomy	12(48.0)	78(56.9)	
Proximal margin(cm)^a^	4(2,6.88)	3(1.5,6.0)	0.287
Distal margin(cm)^b^	4.5(2,9.63)	7(3.30,11.50)	0.096
The number of retrieved LNs	37(37,46.5)	33.0(27,44.5)	0.671
The number of metastatic LNs	0(0.3.5)	2(0,8)	0.014
Retrieved LNs of No. 1-6^c^	22.5(19,29.75)	20(15,26)	0.054
Metastatic LNs of No. 1-6^d^	0(0,3.75)	1(0,4)	0.084
Retrieved LNs of No. 7-9^e^	7.5(5,11)	8(5,10.50)	0.939
Metastatic LNs of No. 7-9^f^	0(0,0.75)	0(0,1.0)	0.433
Retrieved LNs of No. 11, 12a^g^	2(1,3.75)	3(2,6)	0.055
Metastatic LNs of No. 11, 12a^h^	0(0,0)	0(0,0)	0.307

^a^Proximal margin

^b^Distal margin (cm)

^c^Retrieved LNs of No. 1-6

^d^Metastatic LNs of No. 1-6

^e^Retrieved LNs of No. 7-9

^f^Metastatic LNs of No. 7-9

^g^Retrieved LNs of No. 11, 12a

^h^Metastatic LNs of No. 11, 12a: There was one case missing data in the chemotherapy plus immunotherapy group

## Postoperative recovery parameters

There were no significant differences between the two groups in operation time (*p* = 0.197), estimated blood loss (*p* = 0.891), time to first aerofluxus (*p* = 0.349), time to first defecation (*p* = 0.800), time to liquid diet (*p* = 0.233), or length of stay after surgery (*p* = 0.278) (Table 4). All patients received IV-PCA after surgery. The duration of IV-PCA use was shorter in the PCIT group than in the PCT group, with a statistically significant difference [median (IQR), 72 (72, 84) hours vs. 72 (72, 96) hours; *p* = 0.026]. During the hospital stay after surgery, both groups received a comparable amount of supplemental morphine (*p* = 0.881) (Table 4). Neither group showed differences in postoperative pain at 24 h, 48 h, or 72 h after surgery (*p* = 0.375, *p* = 0.601, and *p* = 0.821, respectively) (Table 5).

**Table 4:** Comparison of perioperative recovery parameters between the PCIT group and PCT group

Postoperative recovery parameters	PCIT group (*n*=25)	PCT group (*n*=137)	*p*-value
Operation time (min)	231(185.5,254.5)	205(170,251.50)	0.197
Estimated blood loss (mL)	100(50,175)	100(80,150)	0.891
The first aerofluxus time (days)^a^	3(2,4)	3(3,4)	0.349
The first defecating time (days)^b^	5(4,6)	5(4,6)	0.800
Time to pull drainage (days)^c^	8(6,10)	8(7,10)	0.212
The first time on liquid diets (days)^d^	4(3,5)	5(4,6)	0.233
Length of stay (days)^e^	10(8,14)	11(9,13)	0.278
Using IV-PCA	25(100)	137(100)	1.000
Time of using IV-PCA (h)^f^	72(72,84)	72(72,96)	0.026
Supplementary morphine consumption (mg)	0(0,25.12)	0(0,30.24)	0.881

^a^The first aerofluxus time (days): There was one case missing data in the chemotherapy group

^b^The first defecating time (days): There was one case missing data in the chemotherapy group

^c^Time to pull drainage (days): There were two cases missing data in the chemotherapy group

^d^The first time on liquid diets (days): There were two cases missing data in the chemotherapy group

^e^Time of using IV-PCA (h): There was one case missing data in the chemotherapy group

**Table 5:** Comparison of subjective evaluation of acute pain intensity after surgery between the PCIT and PCT groups

Pain scale	PCIT group (*n*=25)	PCT group (*n*=137)	*p*-value
Postoperative pain at 24h^a^			0.375
Mild [0–3]	20(80.0)	88(64.2)	
Moderate [4–6]	5(20.0)	41(29.9)	
Severe [7–10]	0(0)	7(5.1)	
Postoperative pain at 48h^b^			0.601
Mild [0–3]	18(72.0)	104(75.9)	
Moderate [4–6]	7(28.0)	28(20.4)	
Severe [7–10]	0(0)	4(2.9)	
Postoperative pain at 72h^c^			0.821
Mild [0–3]	16(64.0)	90(65.7)	
Moderate [4–6]	8(32.0)	45(32.1)	
Severe [7–10]	1(4.0)	2(1.5)	

^a, b, c^Postoperative pain at 24h, 48h, 72h: There was one case missing data in the chemotherapy group because of death within 24 hours after surgery

### Schemes and completion rates of postoperative adjuvant chemotherapy

Of the 25 patients in the PCIT group, 16 (64.0%) patients completed postoperative adjuvant chemotherapy. In the PCT group, 81 (59.1%) patients completed postoperative adjuvant chemotherapy. No significant difference was found between the PCIT group and PCT group regarding the rate of completion of postoperative adjuvant chemotherapy (*p* = 0.647). In the PCIT group, six patients received chemotherapy, six patients received chemotherapy plus immunotherapy, and four patients received immunotherapy alone. In the PCT group, 75 patients received chemotherapy, three patients received chemotherapy plus immunotherapy, and three patients received immunotherapy alone (Table 6).

**Table 6:** Comparison of postoperative complications between the PCIT group and PCT group

Postoperative complications	PCIT group (*n*=25)	PCT group (*n*=137)	*p*-value
Complication (%)			0.952
No complication	17(68.0)	94(68.6)	
Complication	8(32.0)	43(31.4)	
Complication classification (%)			0.302
Clavien-Dindo grade I/II	8(32.0)	33(24.1)	
Clavien-Dindo grade III/IV/V	0(0)	10(7.3)	
Perioperative mortality	0(0)	2(1.5)	1.00
Re-hospitalization	16 (64.0)	81 (59.1)	0.647
Anastomotic leak	0(0)	5(3.6)	0.598
All-cause infection	3(12.0)	14(10.2)	1.00
Intra-abdominal infection	1(4.0)	11(8.0)	0.694
Pulmonary infection	2(8.0)	2(1.5)	0.113
Hemorrhage/transfusion	0(0)	2(1.5)	1.000
Lymphatic leak	2(8.0)	4(2.9)	0.232
Pancreatic fistula	0(0)	5(3.6)	0.598

### Postoperative complications

The postoperative complication rate was 32.0% in the PCT group and 31.4% in the PCIT group, with no significant difference (*p* = 0.952). A total of ten severe complications occurred in the PCT group, and none occurred in the PCIT group, with no significant difference (*p* = 0.302). There were two cases of mortality (1.5%) in the PCT group and no cases in the PCIT group. No significant difference was found in perioperative mortality [0% (0/25) in the PCIT group vs. 1.5% (2/137) in the PCT group, *p* = 1.00]. The details are presented in Table 7.

**Table 7:** Content and completion rate of postoperative adjuvant chemotherapy between the PCIT group and PCT group

Postoperative adjuvant chemotherapy	PCIT group (*n*=25)	PCT group (*n*=137)	*p*-value
Completion of postoperative adjuvant chemotherapy			0.647
No	9 (36.0)	56 (40.9)	
Yes	16 (64.0)	81 (59.1)	
Postoperative adjuvant chemotherapy regimen			<0.001
Chemotherapy	6 (24.0)	75 (54.7)	
Chemotherapy plus immunotherapy	6 (24.0)	3 (2.2)	
Immunotherapy alone	4 (16.0)	3 (2.2)	

### Correlation analysis between age and postoperative complications

There was no significant difference in age between the complication group and the noncomplication group [median (IQR) age 63 (54, 69) years in the complication group vs. 63 (52, 68) years in the noncomplication group, *p* = 0.536]. In addition, there was no correlation between age and complications (correlation = 0.160). The details are provided in S1 Table, S2 Table, and S3 Table.

## Discussion

Although immunotherapy has been approved as a standard treatment for metastatic and unresectable GCs, little robust evidence indicates that immunotherapy can be safely used for resectable or potentially resectable GCs before surgery. The present study suggested that the safety and short-term efficacy of gastrectomy after PCIT were comparable to those of PCT in patients with AGC. To the best of our knowledge, this is the first cohort study comparing the safety and efficacy of gastrectomy after PCIT with those of PCT in AGC.

In the present study, the ORR and DCR in the PCT group were comparable to those in the PCIT group. This result suggests that PCIT has efficacy comparable to that of PCT in the treatment of AGC. Furthermore, the number of metastatic LNs in the PCIT group was less than that in the PCT group, with no significant difference in the number of retrieved LNs. A significant difference in the number of patients with ypTNM stage disease between the two groups was also observed, but there was no significant difference in the number of patients with cTNM stage disease. All these results support the idea that PCIT has comparable efficacy to PCT in the treatment of AGC.

In this study, PCIT did not lead to a higher rate of TRAEs than PCT. In addition, the postoperative complication rate in the PCIT group was lower than that previously reported results by our center, ranging from 33.33 to 33.68% in patients who underwent gastrectomy after PCT [7, 10]. This result suggests that gastrectomy could be safely performed by experienced surgeons in patients with AGC after PCIT without increasing the likelihood of postoperative complications or perioperative mortality. Although the results were not statistically significant, complications of Clavien‒Dindo grade III/IV/V occurred only in the PCT group, and all serious complications occurred only in the PCT group. There have been no reports of whether immunotherapy is associated with a decreased risk of serious complications in patients with AGC. The results of this study provide a good basis for further research on the relationship between immunotherapy and serious surgical complications. Furthermore, gastrectomy after PCIT did not increase the operation time, blood loss, time to recovery of gastrointestinal function, or length of hospital stay compared to gastrectomy after PCT. No significant difference was observed between the PCIT and PCT groups in terms of proximal or distal margin lengths or number of retrieved LNs. This study suggests that gastrectomy after PCIT compared to gastrectomy after PCT is a safe treatment method for AGC. A study conducted in Europe proved that PCT could increase the rate of R0 resection and decrease LN metastasis compared to surgery alone [34]. Therefore, the results suggested that gastrectomy after PCIT is a safe and effective method for treating AGC.

By analyzing the baseline characteristics, we found a significant age difference between the two groups. Subsequently, we performed a correlation analysis between age and postoperative complications. No correlation was found between age and postoperative complications. Although there was a significant difference in age between the two groups, it likely did not affect our main results. The duration of IV-PCA use after surgery in the PCIT group was shorter than that in the PCT group, which may be because the PCIT group had younger patients. In a previous meta-analysis of human studies on the impact of age on pain thresholds, it was found that pain thresholds increased with age [35].

There were some limitations in our study. First, the present study was a single-center, retrospective study. Second, there was an unequal number of patients in both groups, with a relatively small sample size in the PCIT group. Finally, the present study included only short-term results and safety data of gastrectomy after PCIT, with no long-term follow-up data.

## Conclusion

This study showed that gastrectomy with D2 or D2 + lymphadenectomy after PCIT had comparable safety and efficacy to PCT in patients with AGC.

## Supplementary Information

**Additional file 1:** **Table S1.** Comparison of age between complication group and none complication group. **Table S2.** Comparison of age between mild complication (Clavien-Dindo grade I/II) group and severe complication (Clavien-Dindo grade III/IV/V) group. **Table S3.** Correlation between age and complication, correlation between age and severe complication.

## Data Availability

The datasets generated and/or analysed during the current study are not publicly available due some patients were enrolled in clinical studies, but are available from the corresponding author on reasonable request.

## Abbreviations

PCIT: Preoperative chemotherapy plus immunotherapy
PCT: Preoperative chemotherapy
AGC: Advanced gastric cancer
TRAEs: Treatment-related adverse events
FDA: U.S. Food and Drug Administration
NMPA: China National Medical Products Administration
MDT: Multidisciplinary team
TMB: Tumor mutational burden
PD-L1 CPS: Programmed death-ligand 1 combined positive score
MSI-H: Microsatellite instability-high
ASA: American Society of Anesthesiology
BMI: Body mass index
CT: Computed tomography
RECIST: Response Evaluation Criteria in Solid Tumors
ORR: Overall response rate
CR: Complete response
PR: Partial response
DCR: Disease control rate
SD: Stable disease
UICC: International Union Against Cancer
TRG: Tumor regression grade
VAS: Visual analog scale
IV-PCA: Intravenous patient-controlled analgesia
OMEs: Oral morphine equivalents
IQR: Interquartile range
NCI-CTCAE: National Cancer Institute Common Terminology Criteria for Adverse Events
PG: Proximal gastrectomy
TG: Total gastrectomy
DG: Distal gastrectomy
LNs: Lymph nodes
LG: Laparoscopic gastrectomy
OG: Open gastrectomy
