# Supplementary material for: Safety and short-term outcomes of gastrectomy after preoperative chemotherapy plus immunotherapy versus preoperative chemotherapy: a retrospective cohort study
Source: BMC Cancer. 2022 Dec 13;22:1306. doi: 10.1186/s12885-022-10272-5 (PMC9749219; doi:10.1186/s12885-022-10272-5)
Supplement: Supplementary file 1 — Additional file 1: Table S1. Comparison of age between complication group and none complication group. Table S2. Comparison of age between mild complication (Clavien-Dindo grade I/II) group and severe complication (Clavien-Dindo grade III/IV/V) group. Table S3. Correlation between age and complication, correlation between age and severe complication. [file 12885_2022_10272_MOESM1_ESM.docx]

**Additional file**

S1 Table Comparison of age between complication group and none complication group

|  | Complication (n=51) | No complication (n=111) | p-value |
| --- | --- | --- | --- |
| Age | 63 (54, 69) | 63 (52, 68) | 0.536 |

S2 Table Comparison of age between mild complication (Clavien-Dindo grade I/II) group and severe complication (Clavien-Dindo grade III/IV/V) group

|  | Mild complication (n=41) | Severe complication (n=10) | p-value |
| --- | --- | --- | --- |
| Age | 59.9 ± 1.78 | 63.5 ± 3.86 | 0.375 |

S3 Table Correlation between age and complication, correlation between age and severe complication

|  | correlation coefficient | p-value |
| --- | --- | --- |
| Age and complication | 0.160 | 0.041 |
| Age and severe complication | 0.065 | 0.414 |
